# Implicit Bias Reflects the Company That Words Keep

**DOI:** 10.3389/fpsyg.2022.871221

**Published:** 2022-06-13

**Authors:** David J. Hauser, Norbert Schwarz

**Affiliations:** ^1^Department of Psychology, Queen’s University, Kingston, ON, Canada; ^2^Mind and Society Center, Department of Psychology, University of Southern California, Los Angeles, CA, United States

**Keywords:** language, implicit bias, semantic prosody, semantic embedding, collocation

## Abstract

In everyday language, concepts appear alongside (i.e., collocate with) related concepts. Societal biases often emerge in these collocations; e.g., female (vs. male) names collocate with art- (vs. science-) related concepts, and African American (vs. White American) names collocate with negative (vs. positive) concepts. It is unknown whether such collocations merely reflect societal biases or contribute to them. Concepts that are themselves neutral in valence but nevertheless collocate with valenced concepts provide a unique opportunity to address this question. For example, when asked, most people evaluate the concept “cause” as neutral, but “cause” is frequently followed by negative concepts (e.g., death, pain, and trouble). We use such semantically prosodic concepts to test the influence of collocation on the emergence of implicit bias: do neutral concepts that frequently collocate with valenced concepts have corresponding implicit bias? In evaluative priming tasks, participants evaluated positive/negative nouns (Study 1) or pictures (Study 2) after seeing verb primes that were (a) strongly valenced (e.g., hate and comfort), (b) neutral in valence but collocated with valenced concepts in corpora (e.g., ease and gain), or (c) neutral in valence and not collocated with valenced concepts in corpora (e.g., reply and describe). Throughout, neutral primes with positive (negative) collocates facilitated the evaluation of positive (negative) targets much like strongly valenced primes, whereas neutral primes without valenced collocates did not. That neutral concepts with valenced collocates parallel the influence of valenced concepts suggests that their collocations in natural language may be sufficient for fostering implicit bias. Societal implications of the causal embedding hypothesis are discussed.

## Introduction

“You shall know a word by the company that it keeps.”

Firth, J. R., 1957.

As Firth (1957) noted, words tend to keep certain company, and the company they keep guides how we understand them (Landauer and Dumais, 1997; Mikolov et al., 2013). In natural language use, the company many words keep is biased in ways that mirror human implicit biases. For instance, female-related words are more likely to occur in family related contexts than are male-related words, whereas male-related words are more likely to occur in science-, technology-, engineering-, and math-related contexts than are female-related words (Caliskan et al., 2017; Lewis and Lupyan, 2020). This observation invited the hypothesis that biased company in language may be a key contributor to implicit bias in human minds (for a review, see Caliskan and Lewis (2020)). Unfortunately, words’ biased company is also related to propositional knowledge, and consciously endorsed beliefs may guide both language use and implicit associations. This ambiguity renders it difficult to determine whether word embeddings exert a causal influence on implicit associations, as Caliskan and Lewis (2020) highlighted.

The present studies avoid this ambiguity by drawing on semantically prosodic words that satisfy two criteria: (1) human raters consider them neutral words that do not have clear positive or negative valence, thus indicating that humans do not endorse these words as being positive or negative, but (2) corpus analyses indicate that these words overwhelmingly keep valenced company. For example, the verb *cause* is rated as neutral in explicit measures of valence but typically occurs alongside negative words (the most common noun collocates within four words to the right are *death, problems, damage, pain, cancer, trouble, concern, disease, effect, harm*; Hauser and Schwarz, 2016). If the company words keep is sufficient to elicit implicit associations, words like *cause* should elicit implicit negative evaluations despite their neutrality on explicit measures of valence. Next, we develop these arguments in more detail and report two experiments, using evaluative priming procedures (Fazio et al., 1986) as a measure of implicit associations.

### Biases in Language and Associative Learning

In everyday language, many words keep specific company (Firth, 1957; Sinclair, 1991). For instance, some words co-occur (i.e., collocate) frequently with specific other words. “Peanut butter” tends to collocate with “jelly”; “crystal” collocates with “clear”; and “pay” collocates with “attention” (iWeb corpus; Davies, 2018). Words can also collocate with sets of words that are all conceptually related, providing clues of semantic relationships. For instance, the word “king” collocates with sets of royalty-related words such as “queen,” “reign,” “prince,” “royal,” and “crown” (iWeb corpus; Davies, 2018).

Similar to collocations that manifest semantic relationships, collocations can also reflect social relationships. In corpora of natural language, many words denoting social categories are biased in ways that mimic associations that are commonly found on implicit measures. Female-related terms collocate more with nursing-related words while male-related terms collocate more with doctor-related words (Bolukbasi et al., 2016). African-American names collocate more with negative words than European-American names; female-related terms collocate more with family related words than male-related terms; and female-related terms collocate less with science- and math-related words than male-related terms (Caliskan et al., 2017; Lewis and Lupyan, 2020). Many of these same biases also appear in child-directed text, such as children’s books, television shows, child-directed speech (Charlesworth et al., 2021; Lewis et al., 2022).

Such findings lend support for the *causal embedding hypothesis*—the idea that the company that words keep in natural language may do more than merely reflect human implicit biases; it may also reinforce them (for a review, see Caliskan and Lewis (2020)). Principles of associative learning provide a theoretical basis for this potential process. Repeated presentation of two stimuli in close proximity nurtures associations, such that activation of one activates the other (Iversen, 1992). This process is key to evaluative conditioning paradigms (Hofmann et al., 2010). Neutral words are evaluated more favorably after repeated subliminal presentation with positive (vs. negative) words (De Houwer et al., 1994). Such processes contribute to automatic associations for concepts that emerge in implicit measures (i.e., measures for which people lack the ability to control the measurement outcome). On the other hand, explicit measures of valence are instruments where people have full control over the measurement outcome. Such measures reflect propositional knowledge that people consciously endorse (Strack and Deutsch, 2004; Gawronski and Bodenhausen, 2006, 2011; Hahn and Gawronski, 2018).

Notably, implicit and explicit measures need not agree on the valence of a concept because they mostly assess different psychological processes (but see De Houwer, 2014, for an example of debates on this idea). People can have an automatic negative association for a concept that manifests in implicit measures even if people would conclude, upon reflection, that there is not anything negative about the concept, as highlighted by Strack and Deutsch (2004; see also, Gawronski and Bodenhausen (2006, 2011)). If the causal embedding hypothesis is true, a concept’s positive/negative implicit associations may reflect its regular appearance with positive/negative words in natural language, even when propositional knowledge may not follow suit. Human biases and implicit associations could indicate the company that words keep.

### Disentangling Linguistic Patterns and Propositional Knowledge

Numerous studies show that word collocations in natural language mirror implicit biases of humans, as observed for gender biases in occupation (Bolukbasi et al., 2016; Caliskan et al., 2017; Lewis and Lupyan, 2020). However, the relationship between natural language collocations and implicit bias is complicated by the reliable observation that propositional knowledge is similarly associated with natural language collocations. For instance, people have preferences for flowers over insects that emerge in explicit measures (Greenwald et al., 1998). These preferences also emerge in natural language collocations; terms denoting types of flowers are more likely to collocate with positively valenced words (than negative words), and terms denoting types of insects are more likely to collocate with negatively valenced words (than positive words; Caliskan et al., 2017). Consistent with the example of insects and flowers, a concept’s explicitly measured valence usually corresponds with the valence of its collocations (Snefjella and Kuperman, 2016). Hence, the company that words keep will usually be indicative of both propositional knowledge about the concept as well as automatic associations the concept elicits.

Therefore, an observed correspondence between a concept’s implicit associations in human minds and its collocations in language does not necessarily signal that linguistic tendencies produce automatic associations (Caliskan and Lewis, 2020). Propositional knowledge may be a “third variable” that produces both linguistic and implicit bias. Accordingly, a sound test of the causal embedding hypothesis needs to avoid linguistic material that is contaminated by valence differences between stimuli in propositional knowledge. Ideally, such a test would be based on concepts that collocate with valenced concepts but lack corresponding valence on explicit measures. Finding that implicit associations for these concepts dovetail with linguistic collocations would increase the plausibility of the causal embedding hypothesis by ruling out the effect of propositional knowledge. To date, such tests have not been reported and propositional knowledge remains a viable alternative mechanism for the correspondence of linguistic embeddings and implicit associations (for a discussion, see Caliskan and Lewis (2020)).

### Semantic Prosody

Semantic prosody provides an avenue for such a test. Some concepts have strong linguistic bias, as indicated by the valence of their collocates, but are nevertheless concepts that people do not endorse, upon reflection, as having positive or negative valence (Hauser and Schwarz, 2016, 2018). Many words with semantic prosody are evaluated as neutral when measured using traditional explicit measures (e.g., semantic differentials or Likert scales) and lack a clearly positive or negative valence. Yet, in natural language use, these words reliably collocate with words of positive or negative valence (Sinclair, 1991; Louw, 1993; Stubbs, 1995; Partington, 2004; Xiao and McEnery, 2006; Hauser and Schwarz, 2016, 2018). As an example, consider the word “cause.” Despite being neutral in explicit ratings of valence (*M* = 5.1; where 1 = unpleasant, 5 = neutral, and 9 = pleasant; Warriner et al., 2013), the 10 most common words that follow “cause” in English are predominantly negative: death, problems, damage, pain, cancer, trouble, concern, disease, effect, and harm (COCA corpus; Davies, 2008). On the other hand “restore” has positive semantic prosody. Despite being nearly neutral in explicit ratings of valence (*M* = 5.9, Warriner et al., 2013) the 10 most common words that follow “restore” in English are mostly positive: order, confidence, balance, power, health, faith, sense, democracy, government, and peace (COCA; Davies, 2008). By drawing on semantically prosodic words that lack valence on explicit measures but collocate with valenced concepts, we can disentangle linguistic associations from propositional knowledge.

If implicit bias reflects linguistic bias, this correspondence should emerge even when people report on explicit measures that the concept is neutral. That is, semantically prosodic words that have valenced collocations in natural language *without* corresponding valence on explicit measures should have corresponding valence on implicit measures. Observing this effect would be consistent with the causal embedding hypothesis, suggesting that human implicit biases can reflect linguistic biases independently from propositional knowledge. As Caliskan and Lewis (2020) noted in their review of this literature, previous research has failed to separate the influence of linguistic bias from the influence of propositional knowledge. The current studies attempt to fill this gap.

### The Current Research

If implicit bias reflects the frequent collocation of concepts in natural language, semantically prosodic words should elicit responses on implicit measures of bias that are consistent with the valence of their linguistic associates. To test this prediction, we first pretest several semantically prosodic words to (i) establish their positive/negative biases in collocations and distributional semantics word embeddings and to (ii) affirm their lack of positive/negative valence on explicit measures. Then, two studies test whether these words have implicit biases in human minds in evaluative priming tasks. If so, this disentangles propositional knowledge from the causal embedding hypothesis and suggests that linguistic bias alone may be sufficient for producing implicit bias.

We report all studies, manipulations, measures, and exclusions. Data, materials, and analysis code are available at https://osf.io/dw46q/.

## Pretesting Summary

We test the implicit bias of semantically prosodic words *via* an evaluative priming task (Fazio et al., 1986). To rule out the influence of propositional knowledge, our pretesting identified words that have biases in natural language, but have no biases in explicit measures. Prior research on semantic prosody (Louw, 1993; Stubbs, 1995; Ellis and Frey, 2009; Hauser and Schwarz, 2016, 2018) has identified words which may potentially fit these parameters. We sampled words from this literature, assessing each word’s valence when rated by human raters and the average valence of each word’s 100 most common collocates. Word valence was measured by normed valence ratings collected by Warriner et al. (2013), whose participants rated the degree to which individual words were pleasant/unpleasant on 9 point scales (1 = unpleasant, 9 = pleasant).^1^ Collocate valence was measured by identifying each word’s 100 most common collocates (with mutual information scores above three, filtering out overly common words) that appeared within four words to the right in COCA (Davies, 2008). Common collocates are similar within three or two words to the right (Sinclair, 1991; Stubbs, 1995). Then, we matched each collocate word with its corresponding valence in Warriner et al. (2013), where 1 = unpleasant and 9 = pleasant, to compute a frequency-weighted average collocate valence.

**FIGURE 1:**
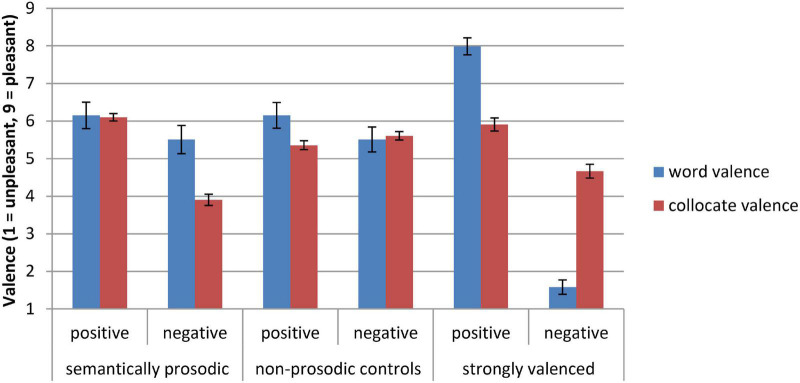
Rated valence and collocate valence of pretested words. Bars indicate ±1 SE.

This piloting identified four words that participants mostly rate as neutral but which keep positive company in everyday language (gain, guarantee, restore, and provide) and identified four words that participants mostly rate as neutral but which keep negative company in everyday language (cause, commit, ease, and peddle). As shown in the first set of bars in Figure 1, on average, the chosen semantically prosodic words had no valence differences in explicit measures, *F*(1, 6) = 3.50, *p* = 0.11, for the effect of semantic prosody. But, the words had biased distributions in natural language, collocating with predominantly positive or negative words. Primes with positive semantic prosody had more positive collocates (*M* = 6.10, *SD* = 0.16) than primes with negative semantic prosody (*M* = 3.90, *SD* = 1.09), *F*(1, 6) = 15.87, *p* = 0.007, *r* = 0.85 for the effect of semantic prosody (see https://osf.io/dw46q/ for additional details).

To create meaningful comparison groups for the semantically prosodic words, we also assembled words that matched for their valence on explicit measures. Using the same measures of word and collocate valence as before, these words did not have valenced company in natural language and received identical word valence ratings as semantically prosodic words (as shown in the middle sets of bars in Figure 1). Thus, they served as matched controls for the valence of semantically prosodic words on explicit measures.

Finally, in order to validate our measure of implicit associations, we also pretested words that had strong valence differences on explicit measures. Using the same measures of word and collocate valence as before, these words were rated by participants in Warriner et al. (2013) as strongly positive/negative (see right sets of bars in Figure 1). For the full analysis code of word stimuli pretesting, see https://osf.io/dw46q/.

We additionally validated the linguistic biases of pretested words *via* measures of semantic embeddings. Semantic embedding models operate on the principal that words that share similar contexts in natural language are semantically associated (Landauer and Dumais, 1997; Mikolov et al., 2013). These models examine large text corpora to uncover patterns in collocations, contexts, and distributive properties in order to map word meanings in multidimensional vector space. Distances between word vectors (i.e., cosine of the angle) can be used as an index of the degree to which words occur in similar contexts, with high numbers representing more similarity. Thus, these models can shed further light on the linguistic biases of words.

To assess linguistic valence biases, we utilized word2vec trained on the google news corpus (Mikolov et al., 2013). We created a valence score for semantically prosodic words and non-prosodic controls by computing the average cosine distance of each verb tense of each word in the group to a set of 25 positive attribute words (e.g., freedom, health, and love) and a set of 25 negative attribute words (e.g., filth, death, and vomit; Greenwald et al., 1998). For each group of words, we calculated the effect size (g) and 95% confidence interval for the difference of similarity to positive vs. similarity to negative words, such that positive (negative) numbers represent association with positive (negative) words and zero represents no association. As a robustness check, we followed the same procedure for 1,000 randomly selected words.

**FIGURE 2:**
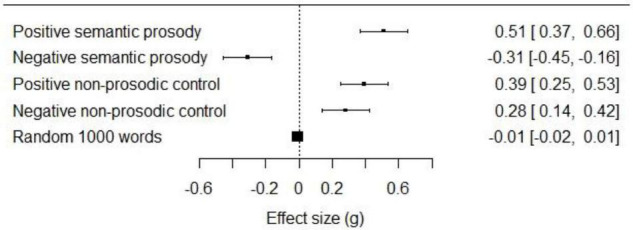
Effect size (g) and 95% CI for valence of words in Study 1 in the semantic embedding model.

As shown in Figure 2, our semantically prosodic words were linguistically biased. Words with positive semantic prosody occurred in similar contexts as positive words, *g* = 0.51, 95% CI [0.37, 0.66], and words with negative semantic prosody occurred in similar contexts as negative words, *g* = -0.31, 95% CI [-0.45, -0.16]. As illustrations, the model identified “cause” as being negative, *g* = -1.05, 95% CI [-1.35, -0.75] and “restore” as being positive, *g* = 0.44, 95% CI [0.16, 0.73].

The measures of linguistic bias did not show the same pattern for non-prosodic control words. While the four positive control words occurred in positive contexts to a similar degree as positive semantically prosodic words, *g* = 0.39, 95% CI [0.25, 0.53], the four negative control words did not occur in negative contexts (instead occurring in predominantly positive contexts), *g* = 0.28, 95% CI [0.14, 0.42]. Thus, non-prosodic control words are not linguistically biased in the same manner as semantically prosodic words, lending validity to the prior pretesting. Finally, a random selection of 1,000 test words showed no significant association with positivity vs. negativity, *g* = 0.01, 95% CI [-0.02, 0.01]. This indicates that our results are not attributable to a biased source of word vector data.

Overall, our pretesting established that (i) we identified semantically prosodic words that are not consciously endorsed as being positive and negative on explicit measures but keep positively and negatively biased company in everyday language and (ii) we have identified matched control words that receive identical valence ratings on explicit measures but do not keep biased company in everyday language. Hence, finding that semantically prosodic words have implicit bias, whereas controls do not, would suggest that language bias corresponds with implicit bias. The following two experiments test this possibility with evaluative priming procedures.

## Study 1

The semantically prosodic words identified in pretesting have no large valence differences on explicit measures. If an implicit measure of valence demonstrates that these words have implicit valence, it would provide evidence that implicit bias reflects linguistic bias, providing further support for the causal embedding hypothesis.

Many different methods exist to assess implicit associations (see the contributions in Wittenbrink and Schwarz (2007)). Although the implicit associations test is currently the dominant method for assessing implicit bias, it is not applicable in the present case because the pretested words lack a readily apparent second dimension, which is required for constructing an IAT (Greenwald et al., 1998). Thus, we assessed implicit valence with another common method, an evaluative priming task (Fazio et al., 1986, 1995). In this task, over several trials, participants evaluate positive and negative target items after briefly seeing a prime concept. Primes that are positive (e.g., flowers) cause participants to more quickly categorize positive (vs. negative) target items, and primes that are negative (e.g., snakes) cause participants to more quickly categorize negative (vs. positive) target items.

This procedure has been adapted to diagnose automatic reactions to prime stimuli that may not be captured by explicit measures (Fazio et al., 1995). If exposure to a prime facilitates the evaluation of negative (vs. positive) targets, the evaluator must have negative associations with the prime. For instance, if images of Black individuals facilitate the evaluation of negative (vs. positive) targets, the evaluator must have negative associations for Black persons (Fazio et al., 1995). In short, when evaluative priming tasks are used as implicit measure of associations, the pattern of facilitation for targets is diagnostic of the prime’s associations.

In Study 1, participants completed an evaluative priming task by evaluating positive and negative nouns after briefly seeing a prime verb. There were two types of primes (previously described in pretesting): semantically prosodic verbs and non-prosodic control verbs. If implicit bias reflects collocational biases in language, then the primes with positive (negative) semantic prosody should facilitate evaluation of positive (negative) target nouns. If this effect were driven by minor differences in the rated valence of positive vs. negative semantically prosodic primes, then non-prosodic positive vs. negative control primes should also create congruity effects since they have identical rated valences. However, if rated valence is not the driving factor, then congruity effects should not emerge for non-prosodic control primes.

### Method

#### Participants

We collected data from as many undergraduate participants as possible before the end of the semester, planning to meet and hopefully exceed the *N* = 49 needed for 80% power to detect an effect size of η*_*p*_*^2^ = 0.146 (the observed effect size of the prime valence × target valence interaction in Ihmels et al. (2016), Study 1). Seventy-eight college students from a large Midwestern university (age range 18–22; 40 females) participated in exchange for introductory psychology course credit. Analyses were not conducted prior to the conclusion of data collection nor were additional data collected after the analyses that follow.

#### Sensitivity Power Analysis

We conducted sensitivity power analyses using G*Power3.1 (Faul et al., 2009) for the predicted congruity effect. G*Power does not calculate interactions between repeated measures, so we computed a more conservative power analysis by treating one of the repeated measures factors as a between groups factor in the congruity effect interaction. Given our sample size, the minimum effect size for the congruity effect that could be detected at 80% power with α = 0.05 and an average correlation between repeated measures of *r* = 0.83 is *f* = 0.094 (η^2^ = 0.0088).

#### Materials

In the main phase of the experiment, participants saw two types of primes (semantically prosodic vs. non-prosodic controls). For more information on properties of the primes, see the previous pretesting section.

Positive and negative nouns were selected as targets of evaluation. The nouns were identified as being strongly positive or negative according to the rated valence norms of Warriner et al. (2013). The positive nouns were comedy (*M*_*valence*_ = 8.05), joy (*M*_*valence*_ = 8.21), delight (*M*_*valence*_ = 8.21), sunshine (*M*_*valence*_ = 8.14), laughter (*M*_*valence*_ = 8.05), and creativity (*M*_*valence*_ = 7.73). The negative nouns were rapist (*M*_*valence*_ = 1.30), racism (*M*_*valence*_ = 1.48), bigotry (*M*_*valence*_ = 2.24), greed (*M*_*valence*_ = 2.48), insult (*M*_*valence*_ = 2.62), and nightmares (*M*_*valence*_ = 1.79). These target nouns did not differ in their number of letters by valence, *F* < 1, nor in the extremity of their valence, *F* < 1.

Because it has already been established that collocations facilitate processing (McKoon and Ratcliff, 1992), we investigated whether any of these nouns were strong collocates of any of our verb primes. None were. That is, in COCA (Davies, 2008) none of these nouns contained any of our verb primes within their top 100 most frequent verbs found within four words to the left among words with mutual information scores above three (Church and Hanks, 1990). This assured that any congruity effects could not be attributed to specific collocations between pairs of primes and targets.

#### Procedure

Our procedures aligned with best practices recommendations for evaluative priming tasks (Wentura and Degner, 2010). Participants completed a study on word judgments in individual cubicles. They were instructed that they would see a brief centering word, followed by a target word that they were to judge as positive or negative. Responses were made with the P and Q keys on the keyboard, and response option mapping (P = positive and Q = negative, or vice versa) was counterbalanced between participants. Participants completed sixteen practice trials in the first phase of the task, followed by 64 actual trials in the second phase.

The experiment was administered in PsychoPy (Peirce, 2007). All words were presented in large white Ariel font in the center of the screen on a gray background. For each trial, participants first saw a verb prime for 200 ms, followed immediately by a target item. Reaction times to categorize the target item and accuracy were recorded. Inaccurate responses were followed by the word “INCORRECT!!!” appearing in red font for 2 s. A 1 s delay preceded the next trial.

In the 16 practice trials, participants categorized positive and negative nouns that were preceded by strongly positive and negative verb primes. Then, in the 64 actual trials, participants categorized positive and negative nouns that were preceded by verb primes (semantic prosody or control). Each of the sixteen verb primes (eight neutral verbs–four with positive and four with negative semantic prosody; eight control verbs without semantic prosody, matching the valence of the semantically prosodic verbs) was presented four times, twice before a positive noun target and twice before a negative noun target. Item-level prime-target pairings were randomly determined *a priori*. Trial order was randomized. After completion, participants reported their age, gender, and major, and were debriefed.

### Results and Discussion

We removed inaccurate trials (4% of trials), log transformed reaction times to reduce positive skew, and replaced times 2.5 standard deviations beyond the grand latency mean with the log cutoff scores (Meier et al., 2007; Robinson, 2007). We then conducted a 2 (prime type: semantically prosodic, non-prosodic control) × 2 (prime valence: positive, negative) × 2 (target valence: positive, negative) repeated measures analysis of variance on log-transformed reaction times.

If implicit bias reflects linguistic bias, then positive (negative) semantically prosodic primes should facilitate evaluation of positive (negative) targets on this implicit measure of associations. As shown in Table 1, this was the case. Negative targets were evaluated faster when preceded by negative semantically prosodic primes than when preceded by positive semantically prosodic primes, *t*(77) = 2.90, *p* = 0.006, *d* = 0.32, 95% confidence interval for the difference between means [0.009, 0.050] for the simple effect. Likewise, positive targets were evaluated faster when preceded by positive semantically prosodic primes than when preceded by negative semantically prosodic primes, *t*(77) = 2.92, *p* = 0.004, *d* = 0.34, 95% CI [0.013, 0.063] for the simple effect. This congruity effect was reflected in a significant simple 2-way interaction of prime valence and target valence for semantically prosodic primes, *F*(1, 77) = 17.11, *p* < 0.001, 95% CI [0.02, 0.05].

**TABLE 1:** Mean (SD) response latencies in milliseconds to categorize target words in Study 1 as a function of prime type, target word valence, and prime valence.

	Target valence	
Prime type by prime valence	Negative	Positive
**Semantically prosodic primes**		
Negative	659 (138)	655 (138)
Positive	675 (128)	629 (132)
**Non-prosodic control primes**		
Negative	668 (130)	630 (123)
Positive	664 (133)	651 (121)

These results show that explicitly neutral words can have implicit positive/negative associations in human minds when they keep valenced company in everyday language. This supports the causal embedding hypothesis even when controlling for propositional knowledge.

Our design allows for a second test of the role of propositional knowledge. If the obtained congruity effect is due to subtle differences in the rated valence of our positive and negative semantically prosodic primes, similar congruity effects should be observed for our non-prosodic control primes with equivalent valence ratings. As shown in Table 1, no such patterns emerged for these primes. When targets were negative, control prime valence did not influence reaction times, *t*(77) = 0.27, *p* = 0.80 for the simple effect. When targets were positive, they were evaluated faster when preceded by negative primes than when preceded by positive primes, reversing the typical pattern of affective priming effects, *t*(77) = 3.50, *p* < 0.001, *d* = 0.38, 95% CI [0.014, 0.055] for the simple effect. This pattern is reflected in a simple two way interaction of prime valence by target valence, *F*(1, 77) = 6.18, *p* = 0.015, 95% CI [0.004, 0.034].

Thus, congruity effects did not emerge for non-prosodic control primes; negative targets were not impacted by non-prosodic positive vs. negative primes, and positive targets were facilitated by non-prosodic negative vs. positive primes (which is opposite of a congruity effect). This makes it further unlikely that the congruity effect obtained for semantically prosodic primes reflects subtle rated valence differences.

The described simple-interaction effects resulted in a three-way interaction of prime valence, target valence, and prime type: *F*(1, 77) = 22.77, *p* < 0.001, η*_*p*_*^2^ = 0.23, 95% CI [0.02, 0.05]. The remaining effects were of little theoretical interest. Target valence affected reaction times: *F*(1, 77) = 29.72, *p* < 0.001, η*_*p*_*^2^ = 0.28, 95% CI [0.04, 0.08] for the main effect. Prime type also marginally moderated the effect of prime valence: *F*(1, 77) = 3.08, *p* = 0.083, η*_*p*_*^2^ = 0.04, 95% CI [-0.002, 0.031] for the two way interaction. All other main effects, *p*s > 0.30, and two-way interactions, *p*s > 0.19, were not significant.

In summary, concepts that keep valenced company in language can have implicit associations with valence without corresponding valences in explicit measures. This provides initial evidence that implicit bias reflects bias in language. Further, implicit associations with valence were not found for non-prosodic control concepts that had the same rated valence as semantically prosodic concepts. This suggests that the observed effects are not attributable to subtle rated valence differences between primes with positive and negative semantic prosody. Study 2 replicates and extends these findings.

## Study 2

While Study 1 controlled for collocation frequency between primes and targets, verbal materials always come with the risk that reaction times may be influenced by differences in collocation frequency that are difficult to detect in corpora. We avoided this complication in Study 2 by using pictures rather than word as targets of evaluation. Hence, participants in Study 2 were asked to evaluate positive and negative pictures. The evaluation of valenced pictures can be reliably facilitated by congruent word primes (Durso and Johnson, 1979; Bajo, 1988; Biggs and Marmurek, 1990; Glaser, 1992), although the naming of affective pictures is not (Spruyt et al., 2002). If implicit bias reflects bias in language, then semantically prosodic words should facilitate evaluation of affectively congruent target pictures. In addition, Study 2 includes trials with verb primes that receive strong positive/negative ratings of valence by participants in order to replicate standard evaluative priming congruity effects, thus ensuring the validity of our measure of implicit valence.

Participants completed an evaluative priming task (Fazio et al., 1986) by evaluating positive and negative pictures after briefly seeing a prime verb. There were three types of primes, previously identified in pretesting: semantically prosodic, non-prosodic controls, and strongly valenced. If implicit bias reflects bias in language, then primes with positive (negative) semantic prosody should facilitate evaluation of positive (negative) target pictures, replicating their effects from Study 1. We expected that congruity effects should not emerge for non-prosodic control primes, replicating their null effects from Study 1. Finally, we expected to replicate standard congruity effects for strongly valenced primes, such that primes that receive strongly positive (negative) explicit ratings facilitate evaluation of positive (negative) target pictures. This replication would add convergent validity to our implicit measure by demonstrating its sensitivity to capturing valence differences between primes.

### Method

#### Participants

We sought to collect as many subject pool participants as possible before the end of semester, planning to meet and hopefully exceed the minimum of *N* = 38 needed for 80% power to detect a congruity effect of the size observed in Study 1 (η^2^ = 0.185). Ninety college students from a large Midwestern university participated in exchange for introductory psychology course credit. One participant reported struggling with the task and misidentified the valence of 41% of target pictures; removing their data left a final sample of 89 participants (41 female, age range 17–23). Analyses were not conducted prior to the conclusion of data collection nor were additional data collected after analyses that follow.

#### Sensitivity Power Analysis

We conducted a sensitivity power analyses using G*Power3 (Faul et al., 2009) for the predicted congruity effect. G*Power does not calculate interactions between repeated measures, so we computed a more conservative power analysis by treating one of the repeated measures factor as a between groups factor in the congruity effect interaction. Given our sample size, the minimum effect size for the congruity effect that could be detected at 80% power with α = 0.05 and an average correlation between repeated measures of *r* = 0.77 is *f* = 0.102 (η^2^ = 0.0103).

#### Materials

Primes were eight semantically prosodic words, eight non-prosodic control words, and eight strongly valenced words. Primes of each group (strongly valenced, semantically prosodic, control) did not differ in number of letters, *F* < 1, nor frequency in COCA (Davies, 2008), *F* < 1. For more information on properties of the primes, see the previous pretesting section.

Twelve positive (*M*_*valence*_ = 8.21) and twelve negative pictures (*M*_*valence*_ = 4.30) from IAPS were selected as the positive and negative evaluation targets based on the valence norms of Lang et al. (1997).^2^ Positive pictures included images of kittens, puppies, and smiling people while negative pictures included images of snakes, cockroaches, and crying children.

#### Procedure

Participants completed the task in individual cubicles with computers. In each trial, they first saw a brief centering word, followed by a target picture that they categorized as positive or negative. Responses were made with the P and Q keys on the keyboard, and response option mapping (P = positive and Q = negative or vice versa) was counterbalanced between participants.

The experiment was administered in PsychoPy (Peirce, 2007). All prime words were presented in large white Ariel font in the center of the screen on a gray background. For each trial, participants first saw a verb prime for 200 ms, followed immediately by a target picture. Reaction times to categorize the target picture and accuracy were recorded. Inaccurate responses were followed by the word “INCORRECT!!!” appearing in red font for 2 s. A 1 s delay preceded the next trial.

Following 12 practice trials, participants completed 96 experimental trials. In each experimental trial they categorized positive and negative pictures after exposure to a control, strongly valenced (positive or negative), or semantically prosodic verb prime (positive or negative). Each verb prime (24 total) was presented four times, twice before a positive target picture and twice before a negative target picture. Item-level prime-target pairings were randomly determined *a priori*. Trial order was randomized. To prevent fatigue, a brief break separated the first 48 experimental trials from the second 48 experimental trials. After the conclusion of the task, participants reported their age, gender, and major, and were debriefed.

### Results and Discussion

Inaccurate trials (3.4% of trials) were removed, and reaction times were log transformed to reduce positive skew. We replaced reaction times that were 2.5 standard deviations beyond the grand latency mean with the log cutoff scores (Meier et al., 2007; Robinson, 2007). We conducted a 3 (prime type: strongly valenced, semantically prosodic, non-prosodic control) × 2 (prime valence: positive, negative) × 2 (target valence: positive, negative) repeated measures analysis of variance on log-transformed reaction times.

#### Does the Standard Congruity Effect Replicate?

Strongly valenced primes (i.e., primes that had explicit ratings of strong positive/negative valence) replicated the standard congruity effect (top two rows of Table 2). Positive pictures were evaluated faster when preceded by a positive (vs. negative) verb prime, *t*(88) = 7.40, *p* < 0.001, *d* = 0.71, 95% CI [0.054, 0.094] for the simple effect of prime valence, whereas negative pictures were evaluated faster when preceded by a negative (vs. positive) verb prime, *t*(88) = 6.67, *p* < 0.001, *d* = 0.77, 95% CI [0.056, 0.104] for the simple effect of prime valence. This is reflected in a simple two way interaction of prime valence and target valence, *F*(1, 88) = 91.66, *p* < 0.001, 95% CI [0.061, 0.093].

**TABLE 2:** Mean (SD) response latencies in milliseconds to categorize target pictures in Study 2 as a function of prime type, target word valence, and prime valence.

	Target valence	
Prime type by prime valence	Negative	Positive
**Strongly valenced primes**		
Negative	569 (99)	615 (100)
Positive	612 (104)	567 (100)
**Semantically prosodic primes**		
Negative	582 (89)	613 (96)
Positive	628 (100)	576 (102)
**Non-prosodic control primes**		
Negative	573 (90)	592 (89)
Positive	586 (97)	600 (101)

#### Does Implicit Bias Correspond With Bias in Natural Language?

If implicit bias corresponds with biased positive or negative company in everyday language, primes that have little to no valence on explicit measures but which have positive (negative) semantic prosody should facilitate evaluation of positive (negative) pictures. This was the case (middle two rows of Table 2), replicating the core finding of Study 1. Positive pictures were evaluated faster when preceded by a verb prime with positive (vs. negative) semantic prosody, *t*(88) = 5.45, *p* < 0.001, *d* = 0.56, 95% CI [0.038, 0.082] for the simple effect of prime valence. Negative pictures were evaluated faster when preceded by a verb prime with negative (vs. positive) semantic prosody, *t*(88) = 6.89, *p* < 0.001, *d* = 0.72, 95% CI [0.044, 0.080] for the simple effect of prime valence. In short, semantically prosodic primes with little to no valence differences are implicitly valenced, *F*(1, 88) = 60.15, *p* < 0.001, 95% CI [0.045, 0.076] for the simple two way interaction of prime valence and target valence.

#### Accounting for Rated Valence Differences

If the previously observed implicit valence is attributable to trends in rated valence differences between primes with positive and negative semantic prosody, a similar congruity effect should emerge for control primes with equivalent rated valences. This was not the case; *F* < 1 for the simple two-way interaction of prime valence and target valence (see bottom two rows of Table 2). This highlights that the influence of the semantically prosodic verbs is not due to minor differences in rated valence. Rather, it was due to differences in valence of the company that semantically prosodic words keep. Semantically prosodic words that have seemingly no rated valence differences, but appear in clearly positive or negative contexts in everyday language, have biased implicit associations that correspond with the company they keep.

#### Additional Effects

The discussed simple interactions are reflected in a significant three way interaction of prime type, prime valence, and target valence, *F*(2, 176) = 24.29, *p* < 0.001, η*_*p*_*^2^ = 0.237, 95% CI [0.025, 0.054]. Other effects emerged which had less bearing upon theory. Overall, responses to the target pictures were significantly slower following a semantically prosodic prime (*M* = 600 ms) than a strongly valenced prime (*M* = 591 ms) or a control prime (*M* = 588 ms); *F*(2, 176) = 8.78, *p* < 0.001, η*_*p*_*^2^ = 0.09, 95% CI [0.015, 0.041] for the main effect of prime type. No other significant main effects emerged, *F*s < 1. There were also significant two way interactions between prime type and target valence, *F*(2, 176) = 8.27, *p* < 0.001, η*_*p*_*^2^ = 0.09, 95% CI [0.016, 0.041], and between prime valence and target valence, *F*(1, 88) = 109.32, *p* < 0.001, η*_*p*_*^2^ = 0.55, 95% CI [0.067, 0.098]. However, these effects are all qualified by the significant three-way interaction of prime type, prime valence, and target valence outlined previously.

#### Comparing the Implicit Bias of Strongly Valenced and Semantically Prosodic Primes

Do semantically prosodic words have implicit valence that is stronger than or equivalent to strongly valenced words? An additional analysis compared the congruity effects of strongly valenced and semantically prosodic primes in order to investigate the relative size of their effects. We conducted a 2 (prime type: strongly valenced, semantic prosody) × 2 (prime valence: positive, negative) × 2 (target valence: positive, negative) repeated measures analysis of variance on log-transformed reaction times. In this analysis, the three-way interaction of prime type, prime valence, and target valence was not significant, *F*(1, 88) = 2.30, *p* = 0.133, 95% CI [-0.004, 0.027]. This indicates that the size of the congruity effect for semantically prosodic primes did not reliably differ from that of strongly valenced primes.

#### Summary

Bias in language corresponds with implicit bias. Words that have little to no rated valence differences, but frequently appear in valenced contexts in everyday language, displayed biased implicit associations that corresponded to the valence of their collocates. Notably, non-prosodic control words that had equivalent rated valence as semantically prosodic words displayed no biased implicit associations with valence, indicating that implicit associations for semantically prosodic words were not attributable to subtle differences in rated valence. Finally, the amount of implicit bias for semantically prosodic words was comparable to that for words with strong valence on explicit measures. Thus, Study 2 replicates and generalizes the key findings of Study 1 while providing further validation of the implicit valence measure. The findings again support the causal embedding hypothesis that linguistic bias may contribute to implicit bias even people do not endorse words as being positive or negative on explicit measures of valence.

## General Discussion

The detrimental effects of implicit bias have been well-documented (Nosek et al., 2002; Greenwald et al., 2009; Cameron et al., 2012). However, there are recent debates regarding what implicit measures do and do not predict (Kurdi et al., 2019). Nevertheless, the possible sources of implicit bias remain controversial (Payne et al., 2017). One may wonder, for example, how members of minority groups develop an implicit bias against their own group (Nosek et al., 2007), and how children can show implicit bias at an early age (Baron, 2015). Language is likely to play an important role.

As the present results illustrate, biased company in language may be sufficient for producing implicit bias in human minds. Two studies demonstrated that the contexts in which concepts appear give them shades of meaning that may be undetectable on explicit measures but manifest on implicit measures. Words that keep valenced company in text but lack consciously endorsed valence differences facilitated the evaluation of valenced words (Study 1) and pictures (Study 2). Further, our implicit measures replicated standard evaluative priming effects (Fazio et al., 1986), indicating that they were diagnostic of evaluative tendencies. Thus, biased company in language may have fostered implicit associations. This suggests that anyone learning a culture’s language may unwittingly learn that culture’s implicit biases.

Our findings are consistent with the causal embedding hypothesis (Caliskan and Lewis, 2020), suggesting that cultural forces play a significant role in fostering implicit bias. Persistent patterns in how words are used in a culture’s everyday language, including the contexts they appear in, may contribute to implicit biases. We, and others (Garg et al., 2018; Caliskan et al., 2017; Charlesworth et al., 2021; Lewis and Lupyan, 2020), have found micro-level associations between word collocations in cultural corpora (books, news articles, and television transcripts) and implicit associations for those words. These observations dovetail with macro-level findings, such as the observation that the degree to which a country’s language has gendered nouns predicts implicit gender biases in that country (Lewis and Lupyan, 2020). While past research has focused upon person-level factors that could predict implicit bias (Nosek et al., 2007), there is growing evidence that systemic cultural patterns can also reinforce it (for a review, see Payne et al. (2017)).

Importantly, our findings suggest that statements in everyday language need not be blatantly biased in order to reinforce implicit associations. Mere collocations suffice. For instance, linguistic biases that associate males with the profession “doctor” and females with the profession “nurse” may be evoked by gender inequities in text representations. Text that contains more male than female doctors and more female than male nurses would establish collocations that may foster implicit bias. Indeed, corpora of children’s books and child-directed speech tend to have analogous gender-biases in collocations (Charlesworth et al., 2021; Lewis et al., 2022). This suggests that one potential avenue for correcting implicit bias is to make efforts for equal representation of underrepresented groups with counter-stereotypical attributes in text.

A possible alternative explanation for our results deserves discussion. Our findings show that words that lack valence in explicit measures but keep valenced company can have valenced associations on implicit measures. But how can we be confident that semantically prosodic words really do not carry valance that is represented in propositional knowledge? Perhaps people merely hesitate to report their valence when asked to in explicit measures, as has been observed for many other concepts? As numerous studies demonstrate, people are motivated to present themselves in a favorable manner by not reporting attitudes that run counter to injunctive norms (Crosby et al., 1980; Dovidio and Fazio, 1992). This observation prompted the development of numerous methodological improvements, from an emphasis on confidentiality (Tourangeau and Yan, 2007) to the classic bogus pipeline (Sigall and Page, 1971) and the recent development of implicit measures (Wittenbrink and Schwarz, 2007). Note, however, that self-presentation concerns likely do not apply to semantically prosodic words, such as “cause” and “restore.” We know of no injunctive norm that discourages people from rating the valence of non-taboo words that have no relation to social groups or controversial topics. Conditions that necessitate socially desirable responding do not seem to apply here, leading us to consider this an unlikely alternative explanation for the current results.

We interpret our results as providing evidence in support of the causal embedding hypothesis that linguistic bias may be a contributor to implicit bias. However, in our studies, the reverse causal pathway could also apply: implicit bias could create linguistic bias. Indeed, implicit biases can shape how people use language (Maass, 1999). Further, our research did not manipulate the linguistic bias of stimuli and examine the effects on implicit bias. Rather, we leveraged words with naturally occurring linguistic bias and examined their corresponding implicit bias. Thus, it remains possible that previously existing implicit biases toward the words we examined produced their linguistic bias.

It is also possible that the two pathways operate in tandem: linguistic bias spurs implicit bias, and implicit bias colors the language people use when they write/discuss those concepts. These processes are difficult to disentangle. As Caliskan and Lewis (2020) suggested, designs looking at whether a culture’s linguistic bias precedes that culture’s implicit bias may be necessary. However, the current research does suggest that a concept’s linguistic bias and implicit bias correspond even when people do not consciously endorse the concept as valenced.

Additionally, the current studies investigated the implicit associations with valence for words that lack valence on explicit measures. However, word norming data collected by other researchers on separate samples of participants (Warriner et al., 2013) was used as the source of explicit valence norms. Future studies may provide stronger evidence by assessing valence for these words using both explicit and implicit measures administered to the same sample of participants.

As Firth (1957) noted, we know words by the company they keep. When words keep biased company in language, their collocations may create congruent implicit associations that appear in human minds. This is even the case when bias cannot be detected with explicit measures. These findings suggest that biased collocations in language may be sufficient for creating and perpetuating implicit bias.

## Data Availability Statement

The datasets presented in this study can be found in online repositories. The names of the repository/repositories and accession number(s) can be found below: https://osf.io/dw46q/.

## Ethics Statement

The studies involving human participants were reviewed and approved by the University of Michigan IRB. The patients/participants provided their written informed consent to participate in this study.

## Author Contributions

DH conceptualized and conducted the studies, analyzed the data, and wrote the first draft of the manuscript, upon which NS provided substantial revisions. Both authors contributed to the article and approved the submitted version.

## Conflict of Interest

The authors declare that the research was conducted in the absence of any commercial or financial relationships that could be construed as a potential conflict of interest.

## Publisher’s Note

All claims expressed in this article are solely those of the authors and do not necessarily represent those of their affiliated organizations, or those of the publisher, the editors and the reviewers. Any product that may be evaluated in this article, or claim that may be made by its manufacturer, is not guaranteed or endorsed by the publisher.

## Footnotes

1 There are several options available for pretesting the rated valence of word stimuli, and the methodological choice may impact the outcome. We chose to rely upon the well-established valence norms of Warriner et al. (2013) rather than ask participants in our studies to rate the valence of word stimuli in order to prevent explicit ratings of word valence and implicit measures of word valence from potentially contaminating each other, as is often an issue when incorporating manipulation checks within an experimental design (Hauser et al., 2018).

2 IAPS pictures: 1460, 1463, 1710, 1750, 2040, 2070, 2091, 2340, 2550, 5760, 7330, 8501, 1030, 1050, 1070, 1120, 1201, 1275, 1300, 1930, 2120, 2800, 6250, 6350.

## References

[B1] Bajo M. T. (1988). Semantic facilitation with pictures and words. *J. Exp. Psychol. Learn. Mem. Cogn*. 14 579–589. 10.1037//0278-7393.14.4.579 2972797

[B2] Baron A. S. (2015). Constraints on the development of implicit intergroup attitudes. *Child Dev. Perspect.* 9 50–54.

[B3] Biggs T. C. Marmurek H. H. (1990). Picture and word naming: Is facilitation due to processing overlap? *Am. J. Psychol.* 103 81–100.

[B4] Bolukbasi T. Chang K. W. Zou J. Y. Saligrama V. Kalai A. T. (2016). Man is to computer programmer as woman is to homemaker? Debiasing word embeddings. *Adv. Neural Inf. Process. Syst.* 29 4349–4357.

[B5] Caliskan A. Bryson J. J. Narayanan A. (2017). Semantics derived automatically from language corpora contain human-like biases. *Science* 356 183–186. 10.1126/science.aal4230 28408601

[B6] Caliskan A. Lewis M. (2020). Social biases in word embeddings and their relation to human cognition. *PsyArXiv* [Preprint]. 10.31234/osf.io/d84kg

[B7] Cameron C. D. Brown-Iannuzzi J. L. Payne B. K. (2012). Sequential priming measures of implicit social cognition: a meta-analysis of associations with behavior and explicit attitudes. *Pers. Soc. Psychol. Rev.* 16 330–350. 10.1177/1088868312440047 22490976

[B8] Charlesworth T. E. Yang V. Mann T. C. Kurdi B. Banaji M. R. (2021). Gender stereotypes in natural language: word embeddings show robust consistency across child and adult language corpora of more than 65 million words. *Psychol. Sci.* 32 218–240. 10.1177/0956797620963619 33400629

[B9] Church K. W. Hanks P. (1990). Word association norms, mutual information, and lexicography. *Comput. Linguist.* 16 22–29.

[B10] Crosby F. Bromley S. Saxe L. (1980). Recent unobtrusive studies of Black and White discrimination and prejudice: a literature review. *Psychol. Bull.* 87 546.

[B11] Davies M. (2008). *The Corpus of Contemporary American English: 450 Million Words, 1990-present.* Available online at: https://www.english-corpora.org/coca/ (accessed May 26, 2022).

[B12] Davies M. (2018). *The iWeb Corpus.* Available online at: https://www.english-corpora.org/iWeb/ (accessed May 26, 2022).

[B13] De Houwer J. (2014). A propositional model of implicit evaluation. *Soc. Pers. Psychol. Compass* 8 342–353.

[B14] De Houwer J. Baeyens F. Eelen P. (1994). Verbal evaluative conditioning with undetected US presentations. *Behav. Res. Ther.* 32 629–633. 8085991 10.1016/0005-7967(94)90017-5

[B15] Dovidio J. F. Fazio R. H. (1992). “New technologies for the direct and indirect assessment of attitudes,” in *Questions about Questions: Inquiries into the Cognitive Bases of Surveys*, ed. Tanur J. (New York, NY: Russell Sage Foundation), 204–237.

[B16] Durso F. T. Johnson M. K. (1979). Facilitation in naming and categorizing repeated pictures and words. *J. Exp. Psychol. Hum. Learn. Mem.* 5 449–459.

[B17] Ellis N. C. Frey E. (2009). “The psycholinguistic reality of collocation and semantic prosody,” in *Formulaic Language Typological Studies in Language*, eds Corrigan R. Moravcsik E. Ouali H. Wheatley K. (Amsterdam: Benjamin Johnson), 473–497.

[B18] Faul F. Erdfelder E. Buchner A. Lang A. G. (2009). Statistical power analyses using G* Power 3.1: tests for correlation and regression analyses. *Behav. Res. Methods* 41 1149–1160. 10.3758/BRM.41.4.1149 19897823

[B19] Fazio R. H. Jackson J. R. Dunton B. C. Williams C. J. (1995). Variability in automatic activation as an unobtrusive measure of racial attitudes: A bona fide pipeline? *J. Pers. Soc. Psychol.* 69 1013–1027. 10.1037//0022-3514.69.6.1013 8531054

[B20] Fazio R. H. Sanbonmatsu D. M. Powell M. C. Kardes F. R. (1986). On the automatic activation of attitudes. *J. Pers. Soc. Psychol.* 50 229–238. 3701576 10.1037//0022-3514.50.2.229

[B21] Firth J. R. (1957). *Papers in Linguistics.* Oxford: Oxford University Press.

[B22] Garg N. Schiebinger L. Jurafsky D. Zou J. (2018). Word embeddings quantify 100 years of gender and ethnic stereotypes. *Proc. Natl. Acad. Sci. U.S.A*. 115, E3635–E3644. 10.1073/pnas.1720347115 29615513 PMC5910851

[B23] Gawronski B. Bodenhausen G. V. (2006). Associative and propositional processes in evaluation: an integrative review of implicit and explicit attitude change. *Psychol. Bull.* 132 692–731. 16910748 10.1037/0033-2909.132.5.692

[B24] Gawronski B. Bodenhausen G. V. (2011). The associative–propositional evaluation model: theory, evidence, and open questions. *Adv. Exp. Soc. Psychol.* 44 59–127.

[B25] Glaser W. R. (1992). Picture naming. *Cognition* 42 61–105. 1582161 10.1016/0010-0277(92)90040-o

[B26] Greenwald A. G. McGhee D. E. Schwartz J. L. (1998). Measuring individual differences in implicit cognition: the implicit association test. *J. Pers. Soc. Psychol.* 74 1464–1480. 9654756 10.1037//0022-3514.74.6.1464

[B27] Greenwald A. G. Poehlman T. A. Uhlmann E. L. Banaji M. R. (2009). Understanding and using the Implicit Association Test: III. Meta-analysis of predictive validity. *J. Pers. Soc. Psychol.* 97 17–41. 10.1037/a0015575 19586237

[B28] Hahn A. Gawronski B. (2018). “Implicit social cognition,” in *Stevens’ Handbook of Experimental Psychology and Cognitive Neuroscience, Developmental and Social Psychology*, Vol. 4 4th Edn, ed. Wixted J. T. (New York, NY: Wiley), 395–427.

[B29] Hauser D. J. Ellsworth P. C. Gonzalez R. (2018). Are manipulation checks necessary? *Front. Psychol.* 9:998. 10.3389/fpsyg.2018.00998 29977213 PMC6022204

[B30] Hauser D. J. Schwarz N. (2016). Semantic prosody and judgment. *J. Exp. Psychol. Gen.* 145 882–896. 10.1037/xge0000178 27243765

[B31] Hauser D. J. Schwarz N. (2018). How seemingly innocuous words can bias judgment: semantic prosody and impression formation. *J. Exp. Soc. Psychol.* 75 11–18.

[B32] Hofmann W. De Houwer J. Perugini M. Baeyens F. Crombez G. (2010). Evaluative conditioning in humans: a meta-analysis. *Psychol. Bull.* 136 390–421. 10.1037/a0018916 20438144

[B33] Ihmels M. Freytag P. Fiedler K. Alexopoulos T. (2016). Relational integrativity of prime-target pairs moderates congruity effects in evaluative priming. *Mem. Cogn.* 44 565–579. 10.3758/s13421-015-0581-8 26689705

[B34] Iversen I. H. (1992). Skinner’s early research: from reflexology to operant conditioning. *Am. Psychol.* 47 1318–1328.

[B35] Kurdi B. Seitchik A. E. Axt J. R. Carroll T. J. Karapetyan A. Kaushik N. (2019). Relationship between the Implicit Association Test and intergroup behavior: a meta-analysis. *Am. Psychol.* 74 569–586. 10.1037/amp0000364 30550298

[B36] Landauer T. K. Dumais S. T. (1997). A solution to Plato’s problem: the latent semantic analysis theory of acquisition, induction, and representation of knowledge. *Psychol. Rev.* 104 211–240.

[B37] Lang P. J. Bradley M. M. Cuthbert B. N. (1997). *International Affective Picture System (IAPS): Technical Manual and Affective Ratings.* (Gainesville, FL: NIMH Center for the Study of Emotion and Attention), 39–58.

[B38] Lewis M. Cooper Borkenhagen M. Converse E. Lupyan G. Seidenberg M. S. (2022). What might books be teaching young children about gender? *Psychol. Sci.* 33 33–47. 10.1177/09567976211024643 34939508

[B39] Lewis M. Lupyan G. (2020). Gender stereotypes are reflected in the distributional structure of 25 languages. *Nat. Hum. Behav.* 4 1021–1028. 10.1038/s41562-020-0918-6 32747806

[B40] Louw B. (1993). “Irony in the text or insincerity in the writer? The diagnostic potential of semantic prosodies,” in *Text and Technology: In Honour of John Sinclair*, eds Baker M. Francis G. Tognini-Bonelli E. (Philadelphia, PA: John Benjamins), 157–176.

[B41] Maass A. (1999). Linguistic intergroup bias: stereotype perpetuation through language. *Adv. Exp. Soc. Psychol.* 31 79–121.

[B42] McKoon G. Ratcliff R. (1992). Spreading activation versus compound cue accounts of priming: mediated priming revisited. *J. Exp. Psychol. Learn. Mem. Cogn.* 18 1155–1172. 10.1037//0278-7393.18.6.1155 1447546

[B43] Meier B. P. Hauser D. J. Robinson M. D. Friesen C. K. Schjeldahl K. (2007). What’s” up” with God? Vertical space as a representation of the divine. *J. Pers. Soc. Psychol.* 93 699–710. 10.1037/0022-3514.93.5.699 17983295

[B44] Mikolov T. Chen K. Corrado G. Dean J. (2013). Efficient estimation of word representations in vector space. *arXiv* [Preprint]. 10.48550/arXiv.1301.3781

[B45] Nosek B. A. Banaji M. R. Greenwald A. G. (2002). Math = male, me = female, therefore math not = me. *J. Pers. Soc. Psychol.* 83 44–59. 12088131

[B46] Nosek B. A. Smyth F. L. Hansen J. J. Devos T. Lindner N. M. Ranganath K. A. (2007). Pervasiveness and correlates of implicit attitudes and stereotypes. *Eur. Rev. Soc. Psychol.* 18 36–88. 10.1038/oby.2003.142 12972672

[B47] Partington A. (2004). “Utterly content in each other’s company”: semantic prosody and semantic preference. *Int. J. Corpus Linguist.* 9 131–156.

[B48] Payne B. K. Vuletich H. A. Lundberg K. B. (2017). The bias of crowds: how implicit bias bridges personal and systemic prejudice. *Psychol. Inq.* 28 233–248.

[B49] Peirce J. W. (2007). PsychoPy—psychophysics software in Python. *J. Neurosci. Methods* 162 8–13. 10.1016/j.jneumeth.2006.11.017 17254636 PMC2018741

[B50] Robinson M. D. (2007). “Lives lived in milliseconds: using cognitive methods in personality research,” in *Handbook of Research Methods in Personality Psychology*, eds Robins R. W. Fraley R. C. Krueger R. F. (New York, NY: The Guilford Press), 345–359.

[B51] Sigall H. Page R. (1971). Current stereotypes: a little fading, a little faking. *J. Pers. Soc. Psychol.* 18 247–255.

[B52] Sinclair J. (1991). *Corpus, Concordance, Collocation.* Oxford: OUP.

[B53] Snefjella B. Kuperman V. (2016). It’s all in the delivery: effects of context valence, arousal, and concreteness on visual word processing. *Cognition* 156 135–146. 10.1016/j.cognition.2016.07.010 27567162 PMC6461444

[B54] Spruyt A. Hermans D. Houwer J. D. Eelen P. (2002). On the nature of the affective priming effect: affective priming of naming responses. *Soc. Cogn.* 20 227–256. 10.3758/bf03195946 17533884

[B55] Strack F. Deutsch R. (2004). Reflective and impulsive determinants of social behavior. *Pers. Soc. Psychol. Rev.* 8 220–247. 10.1207/s15327957pspr0803_1 15454347

[B56] Stubbs M. (1995). Collocations and semantic profiles: on the cause of the trouble with quantitative studies. *Functions of Language*, Vol. 2 eds Teubert W. Krishnamurthy R. (Philadelphia, PA: John Benjamins), 23–55.

[B57] Tourangeau R. Yan T. (2007). Sensitive questions in surveys. *Psychol. Bull.* 133 859–883. 17723033 10.1037/0033-2909.133.5.859

[B58] Warriner A. B. Kuperman V. Brysbaert M. (2013). Norms of valence, arousal, and dominance for 13,915 English lemmas. *Behav. Res. Methods* 45 1191–1207. 10.3758/s13428-012-0314-x 23404613

[B59] Wentura D. Degner J. (2010). “A practical guide to sequential priming and related tasks,” in *Handbook of Implicit Social Cognition: Measurement, Theory, and Applications*, eds Gawronski B. Payne B. K. (New York, NY: Guilford Press), 95–116.

[B60] Wittenbrink B. Schwarz N. (2007). *Implicit Measures of Attitudes.* New York, NY: Guilford Press.

[B61] Xiao R. McEnery T. (2006). Collocation, semantic prosody, and near synonymy: a cross-linguistic perspective. *Appl. Linguist.* 27 103–129.

